# Exercise transiently increases the density of incipient blood clots in antiplatelet-treated lacunar stroke patients

**DOI:** 10.1186/s12959-024-00604-9

**Published:** 2024-04-05

**Authors:** L. B. Nørregaard, K. A. Wickham, J. S. Jeppesen, N. Rytter, L. C. Christoffersen, L. Gliemann, M. Lawrence, P. A. Evans, C. Kruuse, Y. Hellsten

**Affiliations:** 1 The Department of Nutrition, Exercise and Sports, University of Copenhagen, Copenhagen, Denmark; 2 Environmental Ergonomics Lab, Faculty of Applied Health Sciences, Brock University, St. Catharines, ON Canada; 3 Neurovascular Research Unit, Department of Neurology, Herlev Gentofte Hospital, University of Copenhagen, Copenhagen, Denmark; 4 Welsh Centre for Emergency Medicine Research, Morriston Hospital, Swansea Bay University Health Board, Swansea, UK; 5 Swansea University Medical School, Swansea, UK

**Keywords:** *Lacunar stroke patients*, *Exercise*, *Clot microstructure*, *Coagulation*, *Thrombogenicity*

## Abstract

**Introduction:**

Older individuals and, in particular, individuals at risk of recurrent stroke, may be susceptible to thrombosis when participating in exercise, however, this aspect has not been well investigated.

**Methods:**

Clot microstructure and conventional markers of thrombotic risk were determined in twenty lacunar stroke patients and fifteen healthy age-matched controls before, immediately after and 1 h after a bout of moderate intensity cycling exercise. Data were analyzed using a linear mixed model approach.

**Results:**

At rest, clot microstructure (1.69 ± 0.07 vs. 1.64 ± 0.05, corresponding to a difference of ~ 50% in normalized clot mass; *p* = 0.009) and thrombocyte count (73%; *p* < 0.0001) were higher, and activated partial thromboplastin time was lower (18%; *p* = 0.0001) in stroke patients compared to age-matched controls. Acute exercise increased thrombogenic markers similarly in the two groups: incipient clot microstructure (1.69 ± 0.07 vs. 1.74 ± 0.05; *p* = 0.0004 and 1.64 ± 0.05 vs. 1.71 ± 0.04; *p* < 0.0001, for stroke and controls respectively), plasma fibrinogen (12%; *p* < 0.0001 and 18%; *p* < 0.0001, for stroke and controls respectively) and the combined coagulation factors II, VII and X (*p* = 0.0001 and *p* < 0.0001, for stroke and controls respectively).

**Conclusion:**

The results show that exercise transiently increases the risk of blood clot formation in both stroke patients and controls, however, due to the higher baseline thrombogenicity in stroke patients, the post exercise risk of forming blood clots may be higher in this group.

**Trial registration:**

Registered at ClinicalTrials.gov (NCT03635177).

## Introduction

Stroke is the second most common cause of death worldwide, and strokes accounted for 11% of all deaths in 2019 [1]. Approximately one fifth of all strokes are small vessel occlusion strokes, also called lacunar strokes [2], i.e. caused by a thrombus in a small artery located deep in the brain [3]. Existing epidemiological data indicates that about 6–20% of lacunar stroke patients will have recurrent strokes within the subsequent 1–5 years [4, 5]. Therefore safe and effective preventive interventions are warranted. Regular exercise training, with its many known beneficial effects on the cardiovascular system [6], may be one such intervention.

It is well-established that exercise training is beneficial in protecting against cardiovascular disease development and progression [7]. Accordingly, regular exercise is highly recommended for individuals with established cardiovascular disease as well as those with lifestyle-related risk factors [8, 9]. However, paradoxically, evidence suggests that a single bout of exercise may transiently increase the risk of blood clot formation, even in young, healthy individuals [10, 11]. Older individuals and in particular individuals at risk of recurrent stroke, may be even more susceptible to thrombosis when participating in exercise, but this aspect has not been well investigated. An improved understanding of how exercise acutely modulate thrombogenicity is of critical importance to provide exercise guidelines safe for older individuals susceptible to stroke or at risk of recurrent stroke.

There are several commonly used indicators of thrombogenicity, including several plasma markers and measures of platelet function [12–14]. Research of the specifics in blood rheology has led to the development of a novel, global marker of hemostasis, which provides an indication of the resulting microstructural density of a blood clot, and thereby the potential harm it can cause [15, 16]. The microstructural analysis involves determination of the gel point of fresh whole blood and provides information about the fibrin network and thereby, microstructural density of an incipient clot [15, 16]. The gel point is used to determine fractal dimension of the incipient blood clot and the measure has been shown to be related to the density of the mature blood clot by scanning electron microscopy [15, 17]. Clot microstructure is a very sensitive measurement that can detect instantaneous changes in risk of development of harmful blood clots [10], and high clot microstructural density have been shown in individuals with vascular and inflammatory diseases [18–20]. In the current study, analysis of clot microstructure was combined with plasma markers of thrombogenicity to get a detailed assessment of the clotting profile.

Thus, the aim of this study was to evaluate the effects of a single bout of graded moderate intensity cycling exercise on incipient clot microstructure, and plasma clotting biomarkers, in lacunar stroke patients and in healthy age-matched controls. Based on the risk of recurrence in ischemic stroke patients [5], we hypothesized that a single exercise bout of moderate intensity would induce a greater increase in fractal dimension and clotting biomarkers in stroke patients compared to healthy age-matched controls.

## Methods

### Subjects and ethical approval

Twenty patients recently diagnosed with lacunar stroke (53–84 years) and fifteen healthy age-matched controls (55–77 years) were included in the study (Table 1). However, conventional markers of coagulation (fibrinogen, thrombocytes, APTT, and coagulation factors) were only assessed in eight stroke patients and fifteen control participants.

**Table 1:** Subject characteristics

	Control group	Stroke patients
**General**		
Age (yr)	66 ± 6	71 ± 9
Sex (male/female)	11/4	13/7
BMI (kg m^−2^)	25 ± 3	25 ± 3
Single antiplatelet therapy (clopidogrel)	0	17
Dual antiplatelet therapy (clopidogrel and aspirin)	0	3
**Cardiovascular risk factors**		
Hypertension (medicated)	2	15
Obese (BMI ≥ 30 kg m^−2^)	1	2
Prior strokes	0	5
Current smokers	0	0
Total cholesterol (mmol L^−1^)	5.1 ± 0.8	5.1 ± 0.9
LDL cholesterol (mmol L^−1^)	3.3 ± 0.6	2.9 ± 0.8
HDL cholesterol (mmol L^−1^)	1.5 ± 0.4	1.7 ± 0.5
P-glucose (from HbA1c; mmol L^−1^)	5.9 ± 0.7	6.7 ± 1.6
**Blood parameters**		
Fibrinogen (µmol L^−1^)	7.7 ± 1.1	8.9 ± 1.3
INR	1.1 ± 0.1	1.1 ± 0.1
Erythrocytes (x10^12^ L^−1^)	4.4 ± 0.6	4.6 ± 0.3
Hemoglobin (mmol L^−1^)	8.6 ± 1.2	9.0 ± 0.8
Hematocrit (%)	39.5 ± 5.4	41.8 ± 3.5
Leukocytes (x10^9^ L^−1^)	5.1 ± 1.5	7.2 ± 2.0*
**Performance**		
Cycle ergometer test, final workload (watts; W)	157 ± 48	118 ± 35^*^
Cycle ergometer test, final heart rate (HR; bpm)	131 ± 14	115 ± 30^(*)^

Data is presented as mean ± SD

Baseline characteristics including cardiovascular risk factors, blood parameters and performance measures of 20 stroke patients and 15 control subjects

* denotes a significant difference between groups; *p* < 0.05

(*) denotes a trend towards a difference between groups; *p* = 0.06

The clinical criteria for inclusion of the patients was a recent incidence of ischemic small vessel occlusion stroke (lacunar stroke). The stroke patients were recruited at the Copenhagen University Hospital, Herlev, Stroke Unit, Department of Neurology, Copenhagen, Denmark, and the age-matched controls were recruited through local advertisement and through online portals. Pre-determined inclusion criteria for the stroke patients were: >18 years of age, clinical symptoms of small vessel occlusion stroke and concomitant brain imaging (computed tomography scan (CT)/magnetic resonance imaging (MR)-scan with signs of a relevant ischemic small vessel occlusion lesion within the last 5 years), MR and/or CT verified acute lacunar supratentorial or infratentorial infarct (< 2 cm in diameter in acute phase, 1.5 cm in chronic phase), and no clinically significant carotid stenosis or cardioembolic cause of infarct. None of the participants were diagnosed with thrombocythemia, but the stroke patients received single antiplatelet-treatment (three patients received dual antiplatelet-treatment), as part of a standard treatment procedure for lacunar stroke [21, 22]. Seventeen of the stroke patients received single antiplatelet therapy with 75 mg clopidogrel and three stroke patient received 75 mg clopidogrel and 75 mg aspirin (Table 1). CYP2C19 mutations were measured by a gene test to assess the intra-individual ability for activating a clopidogrel antiplatelet response. Only one of the stroke patients was homozygote for this gene, with possible reduced clopidogrel antiplatelet activity. Pre-determined inclusion criteria for the controls were: men and women between 50 to 80 years of age, BMI < 30 kg∙m^−2^ and physically inactive lifestyle (< 2 h of physical exercise per week). Pre-determined exclusion criteria for both groups included: other chronic diseases (e.g., cancer, heart disease, immune diseases), drug or alcohol abuse, and smoking. The median duration from the clinical diagnosis of stroke onset to patient assessment was 24 days, with a range from 6 to 756 days.

The study was approved by the ethical committee of Copenhagen, Region H (H-16048498), conducted in accordance with the guidelines of the *Declaration of Helsinki* and was registered at ClinicalTrials.gov (NCT03635177). The participants were informed about any risk and discomfort related to participating in the study before they gave their written consent to participate.

### Overall study design

Prior to inclusion in the study, participants went through a medical examination carried out by a medical doctor. For stroke patients the health examination took place at Herlev Hospital, and for the control subjects at the Department of Nutrition, Exercise and Sports, University of Copenhagen. The medical examination included a medical history by the doctor identifying the general state of health and former diseases, a resting ECG measurement, resting blood pressure, and a blood sample for screening of health-related factors, such as anemia, infection, coagulation profile, cholesterol, kidney function, fasting blood sugar and plasma glucose. The recruitment and medical examination of the stroke patients were performed in close connection with a stroke neurologist involved in their stroke treatment.

Before the experimental day, participants were not allowed to drink any beverage containing caffeine or take medicine for 24 h prior (except for the antiplatelet therapy for stroke patients) and participants avoided strenuous exercise for 48 h prior. On the experimental day, participants arrived at the laboratory at least 2 h post-prandial (light meal). Participants rested in a supine position for ~ 30 min and a peripheral venous catheter (18-gauge, 32 mm; 393224; BD Venflon Pro Safety) was placed in the antecubital vein for blood sampling performed before, immediately after, and 1 h after a graded cycle test with a “Talk Test” (see below), for determination of clot microstructure, assessed as fractal dimension (d_f_), conventional markers of coagulation and full blood count.

### Cycling exercise protocol

The exercise protocol was a graded cycle test with a “Talk Test”, which has been verified as being appropriate for patients with lacunar strokes [23]. In brief, the participants performed a brief warm-up at 15 watts for 2 min at 60 rpm on a Monark cycle ergometer (Monark Ergomedic 928E, Vansbro, Sweden), then the resistance was increased 15 watts every minute. During the last 10 s of each stage of the cycling test, the participants were required to recite a simple paragraph in Danish out loud to the researchers. The test was terminated when the participant could no longer repeat the paragraph comfortably, which was based on bystander evaluation and self-assessment. This should result in a similar relative workload. The participants wore continuous heart rate monitors (POLAR Team Pro (Polar Electro Oy), Kempele, Finland) during the test.

### Measurements of biomarkers indicating the susceptibility of thrombosis

#### Blood clot microstructure

To determine the blood clot microstructure, the gel point of an incipient blood clot was determined. The measurement of the gel point was conducted by rheometric analysis on untreated blood, immediately after the blood drawn, as described elsewhere [15, 24–26]. Gel point is defined as the transition from a viscoelastic fluid to a viscoelastic solid [15]. From the analysis of the gel point measurement, fractal dimension (d_f_) of the incipient blood clot can be determined [15]. D_f_ analysis provides information about the fibrin network of the developing *ex-vivo* clot, and thereby provides a functional measure of clot microstructure [15, 27]. In brief, blood was drawn from the antecubital vein into a 9 mL blood collection tube without additive (455001; Greiner Bio-One, Austria). 3.5 mL of fresh unaltered blood was added to two opposite sides of a double concentric geometry of a controlled stress rheometer (AR-G2; TA Instruments, New Castle, USA) together with 2 drops of low-viscosity oil, at the constant temperature of 37^∘^C. Sequential frequency measurements (2, 0.93, 0.43 and 0.2 Hz) were immediately performed after loading of the sample, thereby over time obtaining gel point [15]. From the obtained gel point ($\alpha$) measurement, the clot microstructure was quantified by calculating the corresponding fractal dimension (d_f_) using the following equation [28]:

$$d_f=\left(10\cdot\left(\alpha/90\right)-15\right)\;/\left(2\cdot\left(\alpha/90\right)-6\right)$$

### Blood biomarkers of coagulation

After appropriate filling of vacutainers (3.5 mL, 3.2% Sodium Citrate, BD Vacutainer, Becton Dickinson, USA), fibrinogen, activated partial thromboplastin time (APTT), coagulation factors II + VII + X and the International Normalized Ratio (INR) were analyzed at either the Department of Clinical Biochemistry at Rigshospitalet, using an ACL TOP 550 CTS analyzer (Werfen, Warrington, UK) or the Department of Clinical Biochemistry at Herlev Hospital using a Sysmex CS5100 (Siemens Healthcare, Germany). In addition, after gently inverting the vacutainer, platelet count was measured in citrated blood using either a Sysmex XN-450 (Sysmex, Kobe, Japan) or ADVIA 2120i 3M (Siemens Healthcare, Erlagen, Germany).

### Measurement of a biomarker of fibrinolysis

#### Plasminogen activator inhibitor-1 (PAI-1)

PAI-1 protein levels were analyzed in EDTA plasma by use of ELISA according to the manufacturer’s protocol (ab269373, Abcam, Cambridge, UK).

### Statistical analysis

The calculation of sample size was based on previous data from our laboratory on the primary outcome; fractal dimension after acute exercise with a power value for the analyses of > 0.8. The required sample size was estimated to at least 15 subjects in each group [10]. The statistical analyses were performed using Rstudio (Version 4.1.2, R Foundation for Statistical Computing, Vienna, Austria). Graphs were made using GraphPad Prism (GraphPad Software for Windows, Version 9.3.1., San Diego, CA, USA). Data are reported as mean $\pm$ standard deviation (SD) unless otherwise stated. Differences in baseline subject characteristics (Table 1) and delta changes between post exercise and rest between groups were detected using a two-tailed unpaired t-test. A linear mixed-model approach was used to investigate differences within and between groups for the following parameters: fractal dimension, thrombocytes, fibrinogen, APTT, coagulation factors, and PAI-1. Fixed factors were ‘group’ (Control subjects and Stroke patients) and ‘Intervention’ (rest, immediately post exercise, and 1 h post exercise). Subjects were specified as a repeated factor and identifier of random variation. Differences were identified using the ‘emmeans’ package and Sidak adjusted *p*-values are presented. Model reduction was not performed to avoid potential selective inference, meaning that *post hoc* tests were performed despite non-significant interactions [29]. Normal distribution was confirmed using Q-Q plots. Twenty stroke patients and fifteen control subjects were included, however, conventional markers of coagulation (fibrinogen, thrombocytes, APTT, and coagulation factors) were assessed in eight stroke patients and fifteen control participants (See figure legends). Missing data points are described below. For the cardiovascular risk factor parameters (Table 1) two data points were missing in the stroke group due to difficulties drawing blood (*n* = 18), and for hematocrit and erythrocytes additional two data points were missing (*n* = 16) due to a mistake running the analysis (*n* = 2). For the measurement of fractal dimension, in the stroke group, one data point was missing at rest and post-exercise, and three data points in total were missing at 1 h post-exercise due to difficulties drawing blood. In the control group, two data points were missing 1 h post-exercise due to difficulties drawing blood (*n* = 1) and due to a system error during analysis (*n* = 1). For thrombocyte count, one data point was missing from the stroke patients at rest and post-exercise, due to difficulties drawing blood and due to machine error when running the analysis, and one data point was missing from the control subjects 1 h post-exercise due to difficulties drawing blood. For the measurement of fibrinogen and APTT, one data point was missing for stroke patients 1 h post-exercise and from the control subjects pre-exercise, in both cases due to difficulties drawing blood. For APTT, an additional data point was missing in the control subjects post-exercise. For coagulation factors and INR, one data point was missing from the control subjects pre-exercise difficulties drawing blood. For measurements of PAI-1, two data points were missing from the stroke subjects 1 h post-exercise due to insufficient material. The significance level was set at *p* < 0.05, and trends were reported if *p* < 0.07.

## Results

### Characteristics

The subject characteristics are reported in Table 1.

### Clot microstructural density: assessed by fractal dimension of Gel Point

**Fig. 1:**
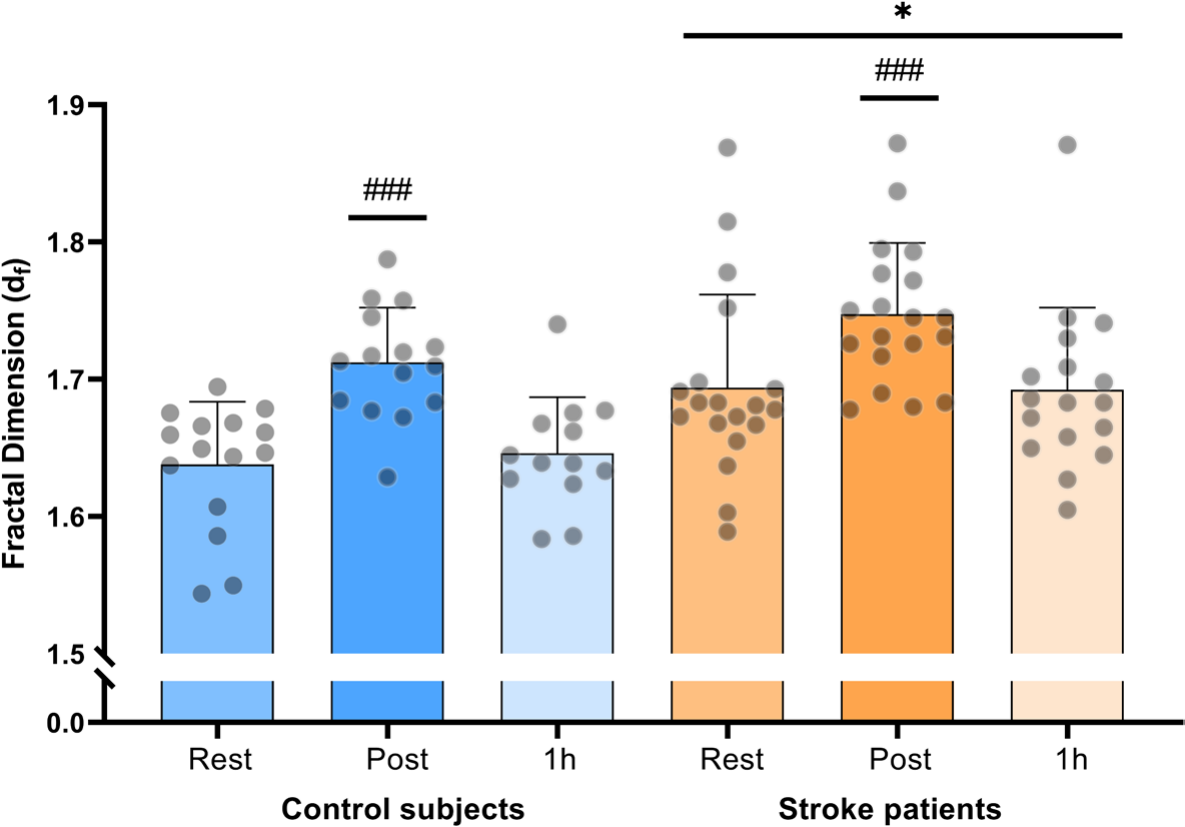
Clot microstructure density, assessed as fractal dimension at rest, immediately post and 1 h post moderate intensity exercise in stroke patients (*n* = 19) and control subjects (*n* = 15). * denotes a difference between groups, ^#^ denotes a difference from pre exercise within group. Significance codes: ’***^/###^’ 0.001 ’*^/#^’ 0.05

There was a main effect of group where stroke patients had higher values of d_f_ compared to control subjects (*p* = 0.011; Fig. 1). Resting d_f_ was higher in the stroke patients compared to control subjects (3%; corresponding to ~ 50% in clot mass; *p* = 0.009). A difference in clot mass of 50% corresponds to a fibrin network with significantly lower fibrin-fibre density and an increase in clot porosity. In response to exercise, both groups presented a similar magnitude of increase in d_f_ (*p* = 0.21). Within the stroke group, d_f_ increased from rest to post moderate intensity exercise (3.6%; *p* = 0.0004; Fig. 1), and d_f_ returned to resting levels 1 h after moderate intensity exercise. In the control group, d_f_ increased from rest to post moderate intensity exercise (4.5%; *p* < 0.0001), and returned to resting levels 1 h after moderate intensity exercise (Fig. 1). One hour after moderate intensity exercise, d_f_ was higher in stroke patients compared to control subjects (*p* = 0.04).

### Full blood thrombocyte count

**Fig. 2:**
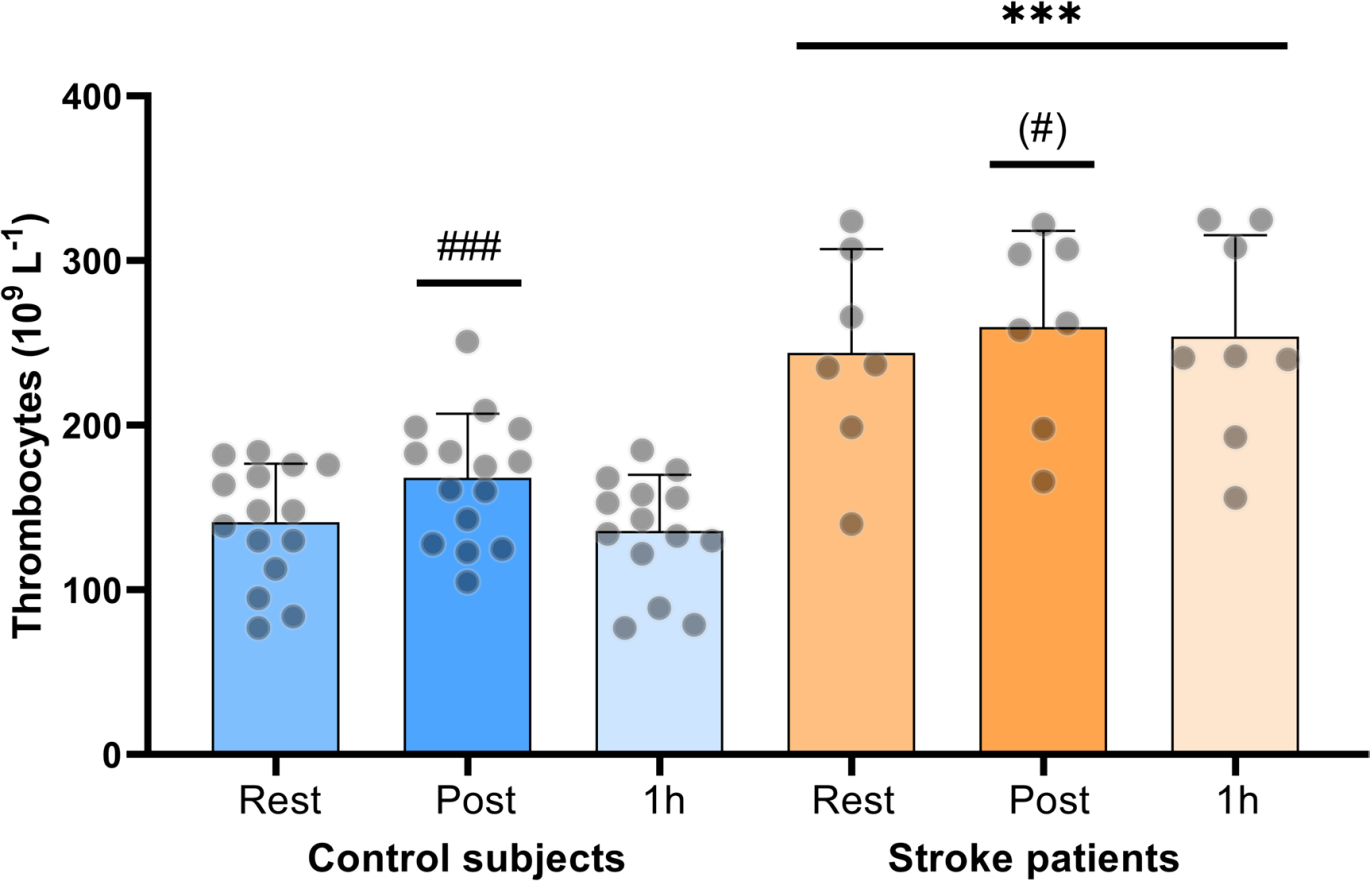
Thrombocyte count at rest, immediately post and 1 h post moderate intensity exercise in stroke patients (*n* = 8) and control subjects (*n* = 15). * denotes a difference between groups, ^#^ denotes a difference from pre-exercise within group. ^(#)^ brackets denote a trend. Significance code: ’***^/###^’ 0.001 ^'(#) ' ^0.065

There was a main effect of group, where stroke patients had a higher thrombocyte count than control subjects (*p* < 0.001), and thrombocyte count was significantly higher in stroke patients compared to control subjects at all time-points (*p* ≤ 0.0001; Fig. 2). Immediately after exercise, the thrombocyte count was higher than at rest in control subjects (19.2%; *p* < 0.0001). In stroke patients there was a tendency towards an increase in thrombocyte count immediately after exercise compared to rest (by 6.4%; *p* = 0.065). The change in thrombocyte count from rest to after exercise was not different between the groups (*p* = 0.35). Thrombocyte count decreased to resting levels 1 h post-exercise in both groups (Fig. 2).

### Plasma biomarkers of coagulation

#### Plasma fibrinogen levels

**Fig. 3:**
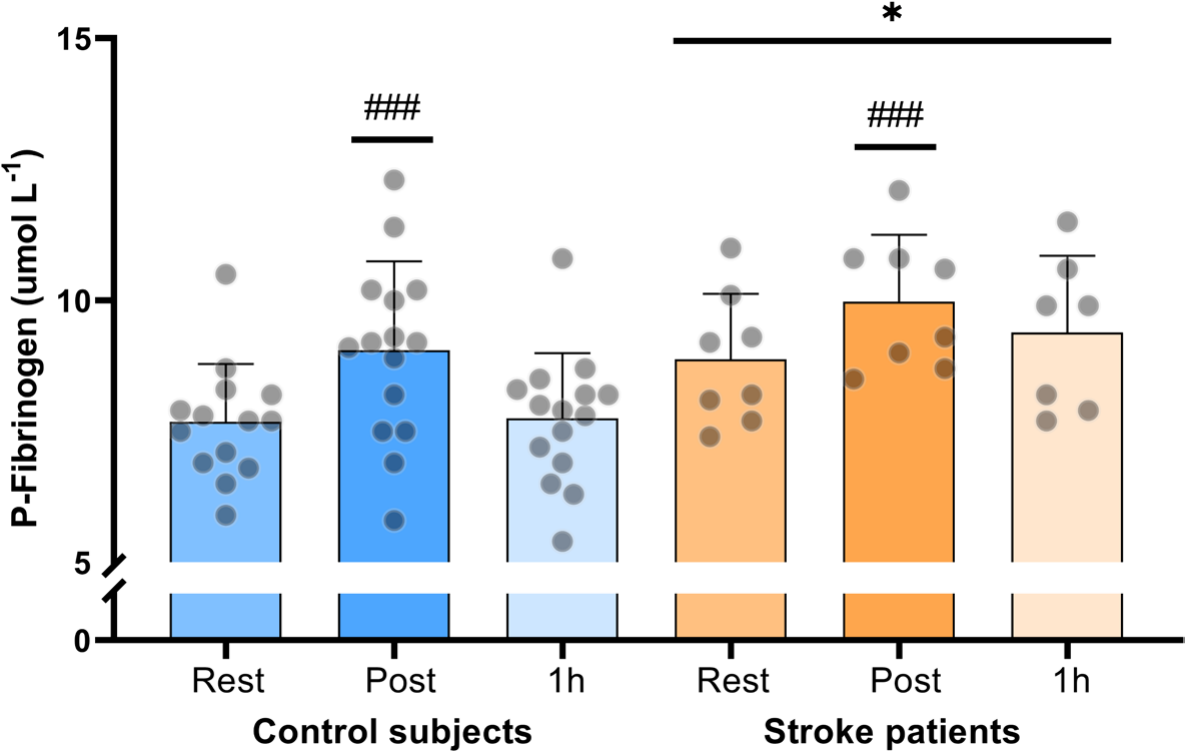
Fibrinogen levels measured at rest, immediately post and 1 h post moderate intensity cycling exercise in stroke patients (*n* = 8) and control subjects (*n* = 15). * denotes a difference between groups, ^#^ denotes a difference from pre-exercise within group. Significance codes: ’***^/###^’ 0.001 ’*^/#^’ 0.05

There was a main effect of group, where stroke patients had higher fibrinogen levels in plasma compared to control subjects (*p* = 0.013). There was no difference in plasma fibrinogen levels between stroke patients and control subjects at rest (*p* = 0.20) or after moderate intensity exercise (*p* = 0.35). There was a tendency towards stroke patients having higher plasma fibrinogen levels 1 h after moderate intensity exercise compared to control subjects (*p* = 0.058). The change in plasma fibrinogen levels with exercise was not different between groups (*p* = 0.56). Immediately after exercise, plasma fibrinogen levels increased compared to rest in both control subjects (*p* < 0.0001) and stroke patients (*p* < 0.0001), and the levels returned to baseline 1 h after exercise in both groups (Fig. 3).

### Plasma APTT

**Fig. 4:**
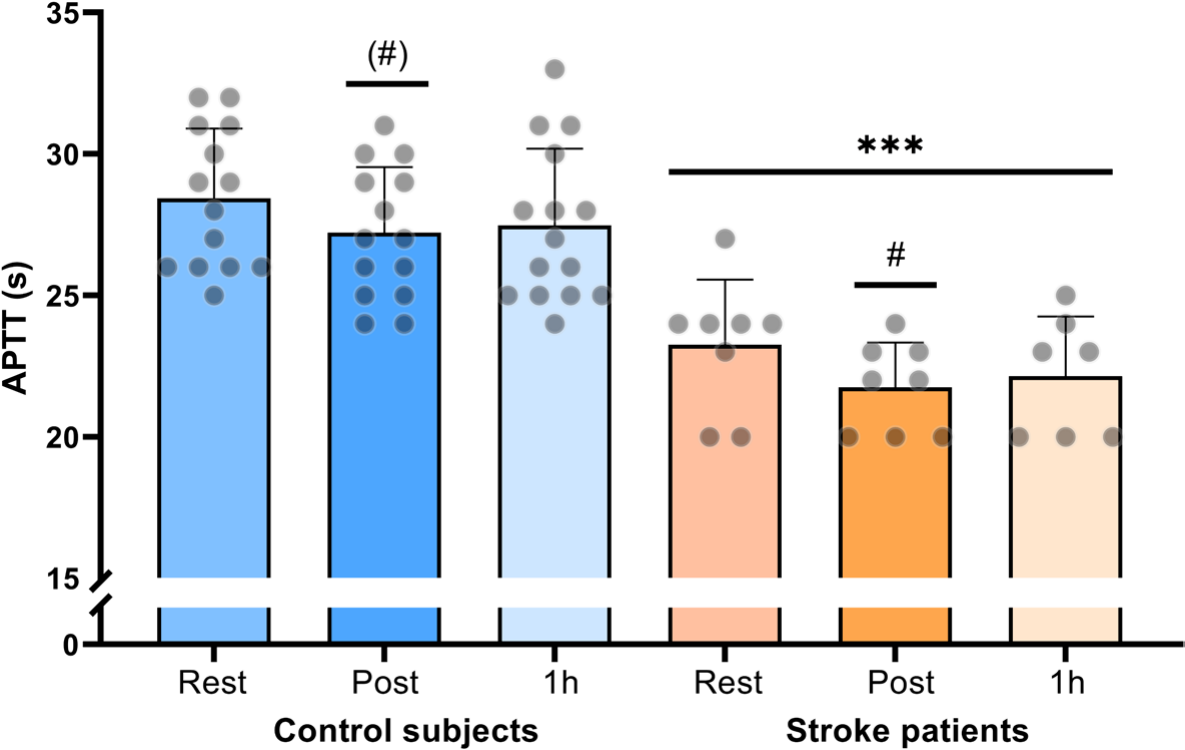
Activated Partial Thromboplastin Time (APTT; s) at rest, immediately post and 1 h post moderate intensity cycling exercise in stroke patients (*n* = 8) and control subjects (*n* = 15). * denotes a difference between groups, ^#^ denotes a difference from pre-exercise within group. ^(#)^ brackets denote a trend. Significance codes: ’***^/###^’ 0.001 ’*^/#^’ 0.05 ^'(#) ' ^0.051

There was a main effect of activated partial thromboplastin time (APTT) between groups, with the stroke patients having lower APTT compared to control subjects (*p* < 0.001; Fig. 4), and APTT was significantly lower in stroke patients compared to control subjects at all time-points (*p* ≤ 0.0001). The change in APTT with exercise was not different between groups (*p* = 0.26). In response to moderate intensity exercise, APTT decreased compared to rest in stroke patients (*p* = 0.01). There was a tendency towards a decrease in APTT in control subjects after moderate intensity exercise compared to rest in control subjects (*p* = 0.051). APTT returned to resting levels in stroke subjects, but not in control subjects (Fig. 4).

### Plasma levels of coagulation factors II + VII + X

**Fig. 5:**
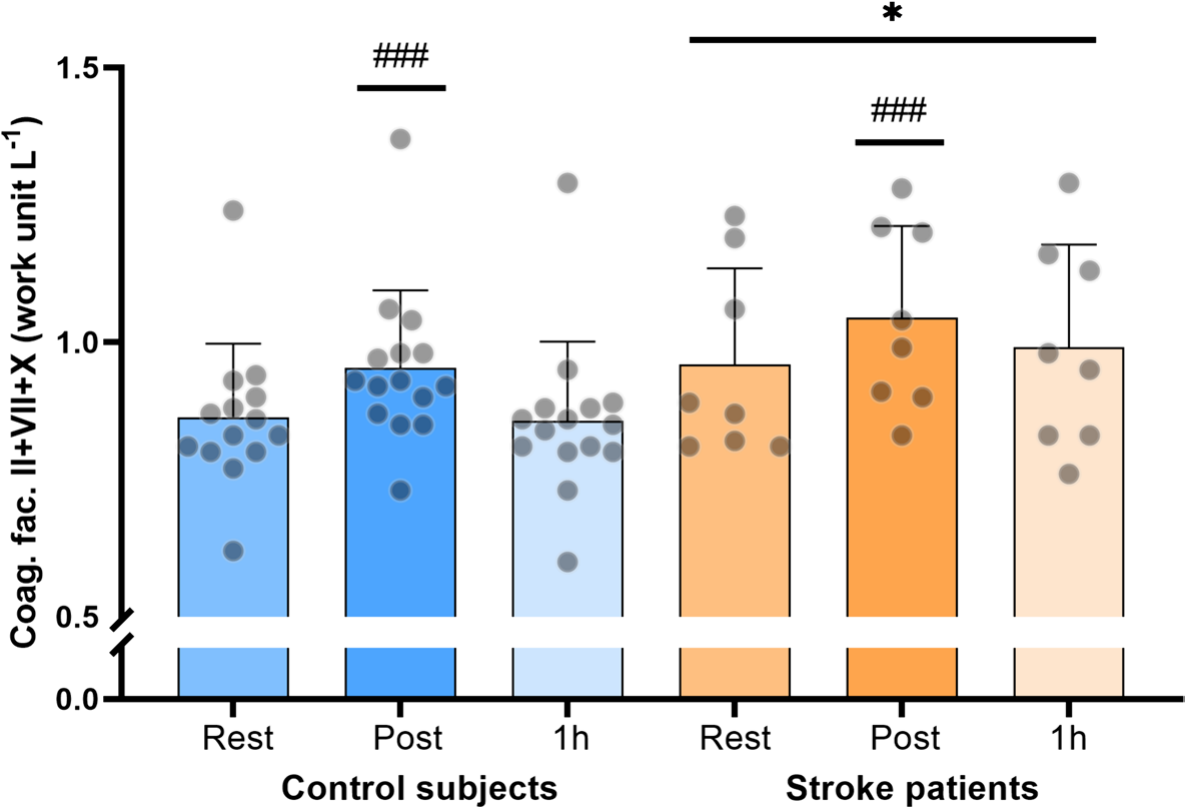
Coagulation factors II + VII + X at rest, immediately post and 1 h post moderate intensity cycling exercise in stroke patients (*n* = 8) and control subjects (*n* = 15). * denotes a difference between groups, ^#^ denotes a difference from pre-exercise within group. Significance code: ’***/^###^’ 0.001 ’*/^#^’ 0.05

There was a main effect of group, where stroke patients presented higher levels of coagulation factors compared to control subjects (*p* = 0.04). The level of plasma coagulation factors was similar between controls and stroke patients at all time-points (*p* > 0.16; Fig. 5). The change in plasma coagulations factors from rest to after exercise was not different between groups (*p* = 0.88). Immediately after exercise, the level of coagulation factors were significantly higher compared to rest in both control subjects (*p* < 0.0001) and stroke patients (*p* = 0.0001). The level of coagulation factors returned to resting levels 1 h after exercise in both groups (Fig. 5).

### Plasma international normalized ratio (INR)

**Fig. 6:**
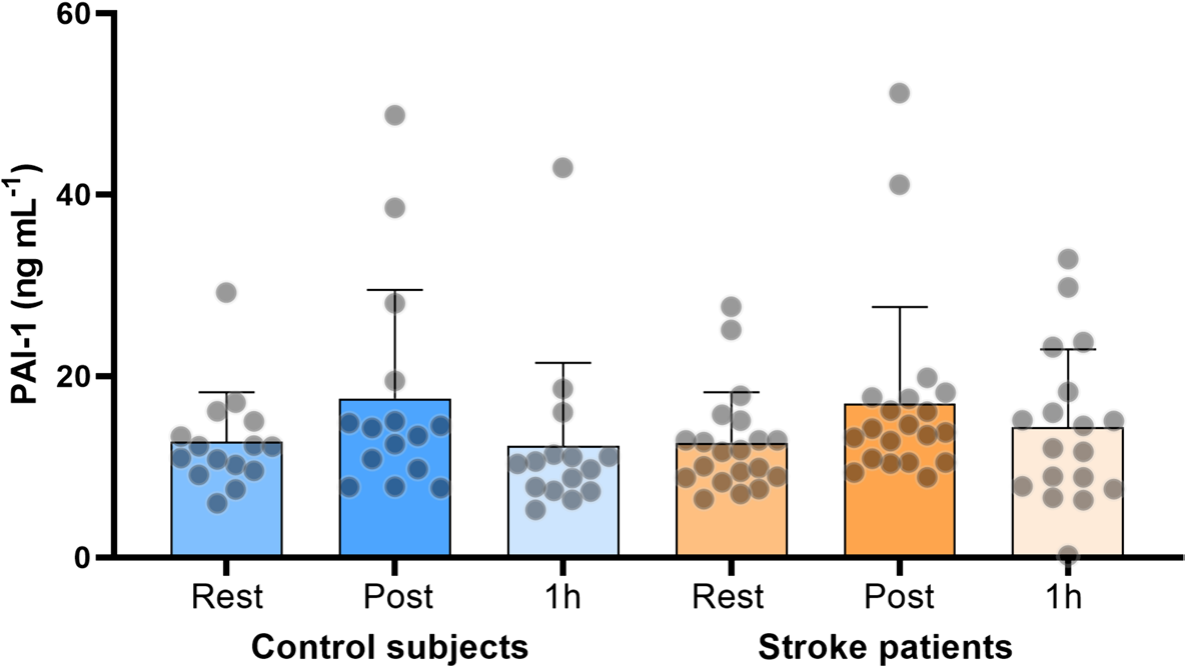
Plasminogen activator inhibitor-1 at rest, immediately post and 1 h post moderate intensity cycling exercise in stroke patients (*n* = 20) and control subjects (*n* = 15)

No difference in plasma INR levels between controls and stroke patients was observed at any time-point (*p* > 0.91; Fig. 6). The change in plasma INR with exercise was not different between groups (*p* = 0.40). Immediately after exercise, INR decreased compared to rest in control subjects (*p* = 0.0012) and returned to resting levels 1 h post-exercise (Fig. 6). In the stroke patients, no difference was observed immediately after exercise compared to rest (*p* = 0.29; Fig. 6).

### Plasminogen activator inhibitor-1 (PAI-1)

Plasma PAI-1 levels were similar in control subjects and stroke patients at all time-points (*p* > 0.90). The change in plasma PAI-1 levels with exercise was not different between groups (*p* = 0.92). In addition, immediately after exercise, no differences were found compared to rest in neither the stroke patients (*p* > 0.41) nor the control subjects (*p* > 0.37; Fig. 6).

## Discussion

The present study evaluated the impact of moderate intensity cycling exercise on clot microstructure, plasma clotting biomarkers and thrombocyte count in lacunar stroke patients and healthy age-matched control subjects. The study aimed to unearth the extent by which a bout of exercise would affect thrombogenicity in lacunar stroke patients. The main findings were that: (I) based on main effects, stroke patients had a more dense clot microstructure, thrombocyte count, plasma fibrinogen level, level of coagulation factors as well as a lower APTT, compared to age-matched control subjects; (II) immediately after a bout of moderate intensity exercise, clot microstructure, plasma fibrinogen, and coagulation factors were higher in both groups with no difference in change between groups; (III) at one hour after exercise, clot microstructure, thrombocyte count, plasma fibrinogen, and coagulation factors had returned to resting levels in both groups.

The measurement of clot microstructure provides an estimate of the density of the incipient blood clot, and represents a sensitive global marker of changes in the risk of arterial thrombosis [27]. By use of this marker in combination with more traditional plasma markers we observed that a brief bout of moderate intensity exercise induced a significant increase in thrombogenicity in both stroke patients and age-matched elderly controls. Although the stroke patients and the control group had similar changes in clot microstructure and clotting biomarkers in the present study, the findings are noteworthy as the stroke patients were on antiplatelet-therapy and performed exercise at a lower workload (118 vs. 157 watts; Table 1) with a consequent lower HR (115 vs. 131 bpm; Table 1) compared to the age-matched control subjects, suggesting that stroke patients may be more sensitive to the thrombogenic risk of acute exercise.

The effect of an acute bout of exercise on incipient clot microstructure has previously been determined in young healthy individuals [10, 11]. The studies showed an increase in clot microstructure density measured directly after an acute bout of both low and maximal intensity cycling exercise [10] or after moderate intensity single-leg knee extensor exercise [11]. The magnitude of change in clot microstructure for both the low intensity and one leg knee extensor exercise in young individuals was somewhat lower than for the present groups of older adults; 0.03 vs. 0.05–0.07, respectively. The immediate interpretation from these data is therefore, that, in terms of throbogenicity, stroke patients are more sensitive to exercise than healthy elderly as well as young individuals. An important observation in the stroke patients was also that some individuals showed large increases in clot microstructure after exercise, suggesting an individualized susceptibility to exercise. In fact, some individuals in the lacunar stroke group had fractal dimension levels above 1.80 (Fig. 1), which are levels observed in serious inflammatory conditions (e.g., in individuals with vascular inflammatory disease) [27]. Based on the present results and the findings that clot microstructure is dependent of intensity [10], exercise programs in stroke patients should preferably be initiated at a low exercise intensity and, importantly, be prescribed on an individual basis including considerations of infections, inflammation and hydration status, to avoid stroke recurrence. Moreover, determination of clot microstructure may provide a sensitive indicator of tolerability of patients to exercise, but also in general for identification of patients who may be prone to recurrent stroke.

Despite a transient increase in clot microstructure and an acute risk of forming blood clots, regular exercise is important, as it has been found to lower thrombogenicity. In elderly females, 8 weeks of exercise training was observed to reduce clot microstructure density [30]. Moreover, well-trained middle-aged males have been shown to have a lower basal platelet reactivity and increased platelet sensitivity for physiological inhibitors during acute exercise than untrained middle-aged males [31]. An additional important point is that the increase in clot microstructure and clotting markers was transient and returned to baseline after only one hour, suggesting the increase in thrombogenicity in association with exercise is relatively brief.

A few studies have investigated the mechanisms underpinning the increase in clot microstructure and plasma clotting markers with acute exercise and have shown an influence of both hemodynamic changes and release of catecholamines [11, 31]. In a study by Lawrence et al. (2018), the effect of hemodynamic changes on clot microstructure was investigated in young males by use of a model activating only a small muscle mass, thereby minimizing the sympathetic drive and release of catecholamines, while increasing muscle blood flow and thereby vascular shear stress. The study revealed an increase in clot microstructure density, pointing to an impact of vascular shear stress on thrombogenicity [11].

In another set of experiments, Lawrence et al. experimentally induced an elevation of circulating catecholamines by arterial infusion of tyramine and found an increase in clot microstructural density [11]. This effect may be related to the platelet activating effect of catecholamines [31, 32], as platelet reactivity is central in the initiation of arterial thrombus formation. The relationship between clot microstructure and catecholamine levels suggests that exercise, which elicits a large sympathetic drive, i.e. intensive exercise and exercise with several muscle groups, may have a great impact on thrombogenicity. This may be particular relevant in stroke patients, as this population has been shown to have elevated catecholamine levels [33]. Although platelet reactivity was not determined in the present study, it has been observed that platelet aggregation is associated with thrombocyte count in patients with coronary artery disease and healthy individuals, even within the normal range of thrombocyte count [34]. In the present group of stroke patients, the thrombocyte count was in the high end of the normal range and 73% higher at rest compared to the control subjects (Fig. 2). Lastly, it is also known that cytokine release (e.g. interleukin 6) increases in response to exercise in an intensity- and duration-dependent manner [35], and cytokine release can influence blood coagulability, thereby increasing the thrombotic risk [36, 37].

How fibrinolysis affects the measure of fractal dimension of the incipient blood clot is unknown, however, a less dense blood clot would present a more permeable fibrin network, enabling dissolution of the clot by the degradation enzyme, plasmin [15]. In the present study, we measured PAI-1, an inhibitor of fibrinolysis, before and after exercise and found no difference in PAI-1 (Fig. 6) between or within groups. Thus it is expected that the observed increase in clot microstructure after exercise in both groups was not influenced by changes in fibrinolysis caused by PAI-1. This is in accordance with previous studies finding no effect of acute exercise on PAI-1 in both healthy individuals and asymptomatic aortic valve stenosis patients [10, 38]. However, clot microstructure could potentially be influenced by other pro- or anti-fibrinolytic compounds that would tip the balance towards either clot formation or clot breakdown.

### Study limitations

One limitation to the study is the lack of assessment of platelet aggregation and FVIII. Such measurements could potentially have contributed to further insight into the mechanisms behind the increased resting clot microstructure in stroke patients, as it has been speculated that platelet activation may predict the risk of stroke recurrence [39] and it has been shown that an increased FVIII is associated with a hypercoagulable state after acute exercise [40]. Another potential limitation was that the thrombogenic markers were analyzed in venous blood only and not arterial blood, however, it has been shown that measures of clot microstructural density are similar in venous and arterial blood obtained before and after exercise [11]. In addition, the effect of acute exercise on fibrinolysis was limited to the measurement of PAI-1, therefore, conclusions regarding the effect of acute exercise on global fibrinolysis in lacunar stroke patients remains elusive. Moreover, there was a slight, but insignificant difference in age between the groups, and as the risk of dehydration could potentially increase with age, this might impact the risk of thrombosis. Therefore, the lack of assessment of hydration status is a limitation to this study. Finally, the standardized talk test was used to provide a safe and easy-to-use exercise protocol. However, it could also have been useful to have a more standardized exercise protocol for lacunar stroke patients in which a specific low intensity protocol is applied for a given time. Future studies should investigate the effect of a specific exercise intensity for a given amount of time in lacunar stroke patients.

### Conclusion and perspectives

Stroke patients were found to form denser blood clots than healthy control subjects at rest, indicating that they have elevated risk of producing potentially harmful blood clots. Moreover, our study shows that an acute bout of moderate intensity exercise transiently increases the propensity to form denser blood clots similarly in both control subjects and antiplatelet-treated stroke patients. Regardless of no differences in delta values between groups, exercise might induce a higher thrombotic risk in lacunar stroke patients compared to control subjects due to the higher thrombogenicity at rest. The observed individual variation in clot microstructure and response suggests that some individuals may be at an elevated risk for stroke recurrence during exercise and since fractal dimension seems to be related to the standard coagulation markers, a pre-assessment of fractal dimension in response to exercise may provide a useful tool in risk assessment when evaluating exercise guidelines. This study provides novel data of risk assessment of recurrence in stroke patients using an easy-to-use exercise protocol based on self-assessment of intensity that can be used in the process of creating safe guidelines for exercise in this patient group. Further studies are required in order to make clear safe exercise recommendations for this patient group.

## Data Availability

The data presented in this study is available upon reasonable request and if in accordance with current General Data Protection Regulation (GDPR) guidelines.

## Abbreviations

APTT: Activated partial thromboplastin time
d_f_: Fractal dimension
PAI-1: Plasminogen activator inhibitor 1
INR: International normalized ratio
CT: Computed tomography scan
MR: Magnetic resonance imaging
BMI: Body mass index
LDL: Low-density lipoprotein
HDL: High-density lipoprotein
ECG: Electrocardiogram
HR: Heart rate
